# Supplementary material for: Earthworms neutralize the influence of components of particulate pollutants on soil extracellular enzymatic functions in subtropical forests
Source: PeerJ. 2023 Aug 3;11:e15720. doi: 10.7717/peerj.15720 (PMC10404396; doi:10.7717/peerj.15720)
Supplement: Supplemental Information 1 [file peerj-11-15720-s001.docx]

**Supplementary material**

**Detailed methods for soil enzyme activity analyses**

Activities of cellobiohydrolase (E.C. 3.2.1.91), β-1,4-glucosidase (E.C. 3.2.1.21), and β-1,4-xylosidase (E.C. 3.2.1.37) were measured using 1.2 mM 4-nitrophenyl-b-D-linked (PNPX) substrates (cellobioside, glucopyranoside, and xylopyranoside) with incubated in the dark at 40 °C for 1.5 h (pH 5.0; 0.2 M Na_2_CO_3_ was added to stop the reaction). 4-Nitrophenyl (PNP) concentrations were quantified by measuring absorbance at 400 nm using a microplate spectrophotometer (Tecan Safire2, Switzerland) in 96-well plates *(Vepsäläinen et al., 2001*). Enzymatic activities are expressed in μ mol PNP h^–1^ g^–1^ soil.

Activity of nitrate reductase (E.C. 1.7.99.4) was measured using 200 m M KNO_3_ solution as a substrate and was incubated at room temperature for 30 min (pH 7.5; NO_2_^–^). Concentrations were determined with a spectrophotometer (JingHua, Shanghai, China) at 520 nm wavelength. Activity was quantified by reference to a calibration curve incubated with soil under the same conditions described and is expressed in μg NO_2_^–^ min^–1^ g^–1^ soil (*Daniel & Curran, 1981*).

Urease (E.C. 3.5.1.5) activity was measured with urea as substrate and was incubated at pH 6.7 (0.2 M phosphate buffer) and 37 °C for 24 h, and absorbance was measured at 578 nm using a spectrophotometer (*Nannipieri et al., 1980*). The enzymatic activity is expressed in mg NH_3_^–^N h^–1^ g^–1^ soil.

Activities of acid phosphatase (E.C. 3.1.3.2) and alkaline phosphatase (E.C. 3.1.3.1) were measured using 0.5% disodium phenyl phosphate solution as substrate and was incubated at 37 °C for 24 h (pH 5.0 for ACP; pH 10.0 for ALP; phenol concentration was determined with a spectrophotometer at 570 nm). Activities were quantified using a calibration curve incubated with soil under the same conditions described and is expressed in mg P h^–1^ g^–1^ soil *(Kandeler, Tscherko & Spiegel, 1999*).

Activities of peroxidase (E.C. 1.11.1.7) and polyphenol oxidase (E.C. 1.10.3.2) activities were measured spectrophotometrically using 50 mL of 25 mM l-3,4-dihydroxy-phenylalanine (L-DOPA) as the substrate with incubation at 28 ℃ for 1 h (pH 5.5). Peroxidase assays plus 10 ml of 0.3% H_2_O_2_ were created before measurement. Activity was quantified by measuring absorbance at 450 nm using a microplate spectrophotometer (Tecan Safire2, Männedorf, Switzerland) in 96-well plates and expressed in units of nmol h^-1^ g^-1^ *(Saiya-Cork, Sinsabaugh & Zak, 2002*).

**Daniel R, Curran M. 1981.** A method for the determination of nitrate reductase. *Journal of Biochemical and Biophysical methods* 4:131–132. DOI: 10.1016/0165-022X(81)90026-9.

**Kandeler E, Tscherko D, Spiegel H. 1999.** Long-term monitoring of microbial biomass, N mineralisation and enzyme activities of a Chernozem under different tillage management. *Biology and fertility of soils* 28:343–351. DOI: 10.1007/s003740050502.

**Nannipieri P, Ceccanti B, Cervelli S, Matarese E. 1980.** Extraction of phosphatase, urease, proteases, organic carbon, and nitrogen from soil. *Soil Science Society of America Journal* 44:1011–1016. DOI: 10.2136/sssaj1980.03615995004400050028x.

**Saiya-Cork KR, Sinsabaugh RL, Zak DR. 2002.** The effects of long term nitrogen deposition on extracellular enzyme activity in an *Acer saccharum* forest soil. *Soil Biology and Biochemistry* 34:1309–1315. DOI: 10.1016/S0038-0717(02)00074-3.

**Vepsäläinen M, Kukkonen S, Vestberg M, Sirviö H, Maarit Niemi R. 2001.** Application of soil enzyme activity test kit in a field experiment. *Soil Biology and Biochemistry* 33:1665–1672. DOI: 10.1016/S0038-0717(01)00087-6.

**Tables**

**Table S1:**

**Changes in abundance and biomass of total earthworms, and abundance and biomass of *Eisenia fetida* due to the addition of particulate components (PC) and earthworms (*E. fetida*) (E) in mesocosms of deciduous and coniferous forests.**

Chi^2^- and P-values based on generalized linear models.

|  |  | Deciduous | |  | Coniferous | |
| --- | --- | --- | --- | --- | --- | --- |
|  | Df | Chi^2^ | P |  | Chi^2^ | P |
| **Total abundance** |  |  |  |  |  |  |
| Particulate components (PC) | 3 | 3.18 | 0.364 |  | **11.96** | **0.008** |
| Earthworms (E) | 1 | **19.83** | **<0.001** |  | **14.14** | **<0.001** |
| PC × E | 3 | 0.86 | 0.835 |  | 1.43 | 0.698 |
| **Total biomass** | |  |  |  |  |  |
| Particulate components (PC) | 3 | 5.59 | 0.134 |  | 20.27 | <0.001 |
| Earthworms (E) | 1 | **27.04** | **<0.001** |  | **19.99** | **<0.001** |
| PC × E | 3 | 0.14 | 0.987 |  | 2.42 | 0.490 |
| ***Eisenia* abundance** | |  |  |  |  |  |
| Particulate components (PC) | 3 | 1.11 | 0.775 |  | 4.26 | 0.234 |
| Earthworms (E) | 1 | **18.02** | **<0.001** |  | **17.81** | **<0.001** |
| PC × E | 3 | 1.65 | 0.648 |  | 5.44 | 0.142 |
| ***Eisenia* biomass** | |  |  |  |  |  |
| Particulate components (PC) | 3 | 2.98 | 0.394 |  | 4.14 | 0.246 |
| Earthworms (E) | 1 | **17.67** | **<0.001** |  | **15.59** | **<0.001** |
| PC × E | 3 | 0.69 | 0.875 |  | 4.07 | 0.254 |

**Table S2:**

**Results (SS, R^2^, F- and P-values) of permutational multivariate analysis of variance on the effects of particulate components (N, Na, PAHs), earthworms (with, without), mesh size (fine, coarse) and time (70, 140, 210, 280, 365 days) on changes in soil total enzyme activities in deciduous and coniferous forests.**

| **Factors** | Deciduous | | | | |  | Coniferous | | | | |
| --- | --- | --- | --- | --- | --- | --- | --- | --- | --- | --- | --- |
|  | df | SS | R^2^ | F | P |  | df | SS | R^2^ | F | P |
| Particulate components (PC) | 3 | 0.07 | 0.02 | 2.38 | **0.042** |  | 3 | 0.23 | 0.03 | 5.39 | **0.001** |
| Earthworm (E) | 1 | 0.01 | 0.00 | 0.73 | 0.428 |  | 1 | 0.00 | 0.00 | 0.08 | 0.863 |
| Mesh size (M) | 1 | 0.11 | 0.03 | 10.6 | **0.001** |  | 1 | 0.08 | 0.01 | 5.23 | **0.018** |
| Time (T) | 4 | 0.33 | 0.08 | 8.24 | **0.001** |  | 4 | 1.08 | 0.16 | 18.83 | **0.001** |
| PC × E | 3 | 0.09 | 0.02 | 3.16 | **0.012** |  | 3 | 0.01 | 0.00 | 0.25 | 0.893 |
| PC × M | 3 | 0.01 | 0.00 | 0.33 | 0.880 |  | 3 | 0.12 | 0.02 | 2.85 | **0.023** |
| E × M | 1 | 0.00 | 0.00 | 0.24 | 0.689 |  | 1 | 0.00 | 0.00 | 0.19 | 0.761 |
| PC × T | 12 | 0.11 | 0.03 | 0.94 | 0.521 |  | 12 | 0.48 | 0.07 | 2.77 | **0.002** |
| E × T | 4 | 0.01 | 0.00 | 0.25 | 0.966 |  | 4 | 0.03 | 0.00 | 0.53 | 0.774 |
| M × T | 4 | 0.48 | 0.12 | 12.08 | **0.001** |  | 4 | 0.66 | 0.10 | 11.44 | **0.001** |
| PC × E × M | 3 | 0.09 | 0.02 | 3.04 | **0.010** |  | 3 | 0.02 | 0.00 | 0.44 | 0.756 |
| PC × E × T | 12 | 0.20 | 0.05 | 1.72 | **0.041** |  | 12 | 0.09 | 0.01 | 0.50 | 0.943 |
| PC × M × T | 12 | 0.10 | 0.02 | 0.83 | 0.641 |  | 12 | 0.43 | 0.06 | 2.51 | **0.001** |
| E × M × T | 4 | 0.02 | 0.00 | 0.40 | 0.867 |  | 4 | 0.01 | 0.00 | 0.12 | 0.990 |
| PC × E × M × T | 12 | 0.22 | 0.05 | 1.87 | **0.024** |  | 12 | 0.07 | 0.01 | 0.38 | 0.982 |
| Residual | 230 | 2.28 | 0.55 |  |  |  | 240 | 3.45 | 0.51 |  |  |
| Total | 309 | 4.13 | 1 |  |  |  | 319 | 6.76 | 1 |  |  |

**Table S3:**

**F- and P-values of linear mixed-effects models on the effects of particulate components (N, Na, PAHs), earthworms (with, without), mesh size (fine, coarse) and time (70, 140, 210, 280, 365 days) on changes in Z scores of soil enzyme activities of C, N, P cycling and oxidases (C enzymes, N enzymes, P enzymes, O enzymes) in the deciduous forest.**

C enzymes include β-1,4-glucosidase, β-1,4-xylosidase and cellobiohydrolase, N enzymes include nitrate reductase and urease, P enzymes include acid and alkaline phosphatases, and O enzymes include peroxidase and polyphenol oxidase; df, numerator and denominator degrees of freedom.

| **Factors** | df | C enzymes | | N enzymes | | P enzymes | | O enzymes | |
| --- | --- | --- | --- | --- | --- | --- | --- | --- | --- |
|  |  | F | P | F | P | F | P | P | P |
| (Intercept) | *1,184* | 0.00 | 1.000 | 0.00 | 1.000 | 0.00 | 1.000 | 0.00 | 1.000 |
| Particulate components (PC) | *3,23* | 7.99 | **0.001** | 16.19 | **<0.001** | 2.31 | 0.103 | 10.43 | **<0.001** |
| Earthworms (E) | *1,23* | 1.74 | 0.201 | 2.63 | 0.118 | 2.06 | 0.164 | 0.66 | 0.427 |
| Mesh size (M) | *1,23* | 0.23 | 0.637 | 7.11 | **0.014** | 0.25 | 0.625 | 142.13 | **<0.001** |
| Time (T) | *4,184* | 54.14 | **<0.001** | 104.25 | **<0.001** | 26.41 | **<0.001** | 316.15 | **<0.001** |
| PC × E | *3,23* | 6.16 | **0.003** | 5.93 | **0.004** | 0.85 | 0.481 | 3.52 | **0.031** |
| PC × M | *3,23* | 0.36 | 0.783 | 0.96 | 0.427 | 2.46 | 0.088 | 12.32 | **<0.001** |
| E × M | *1,23* | 0.08 | 0.786 | 0.30 | 0.592 | 2.85 | 0.105 | 7.29 | **0.013** |
| PC × T | *12,184* | 2.55 | **0.004** | 3.86 | **<0.001** | 2.72 | **0.002** | 28.97 | **<0.001** |
| E × T | *4,184* | 1.87 | 0.118 | 2.40 | 0.052 | 0.50 | 0.734 | 3.39 | **0.011** |
| M × T | *4,184* | 9.29 | **<0.001** | 57.72 | **<0.001** | 22.16 | **<0.001** | 118.38 | **<0.001** |
| PC × E × M | *3,23* | 2.54 | 0.082 | 4.13 | **0.018** | 0.43 | 0.736 | 6.06 | **0.003** |
| PC × E × T | *12,184* | 1.55 | 0.108 | 2.29 | **0.010** | 3.38 | **<0.001** | 3.97 | **<0.001** |
| PC × M × T | *12,184* | 1.04 | 0.414 | 0.97 | 0.477 | 5.12 | **<0.001** | 13.79 | **<0.001** |
| E × M × T | *4,184* | 1.57 | 0.185 | 1.33 | 0.259 | 1.35 | 0.253 | 8.59 | **<0.001** |
| PC × E × M × T | *12,184* | 1.12 | 0.346 | 0.87 | 0.578 | 1.53 | 0.118 | 4.57 | **<0.001** |

**Table S4:**

**F- and P-values of linear mixed-effects models on the effects of particulate components (N, Na, PAHs), earthworms (with, without), mesh size (fine, coarse) and time (70, 140, 210, 280, 365 days) on changes in Z scores of soil enzyme activities of C, N, P cycling and oxidases (C enzymes, N enzymes, P enzymes, O enzymes) in the coniferous forest.**

C enzymes include β-1,4-glucosidase, β-1,4-xylosidase and cellobiohydrolase, N enzymes include nitrate reductase and urease, P enzymes include acid and alkaline phosphatases, and O enzymes include peroxidase and polyphenol oxidase; df, numerator and denominator degrees of freedom.

| **Factors** | df | C enzymes | | N enzymes | | P enzymes | | O enzymes | |
| --- | --- | --- | --- | --- | --- | --- | --- | --- | --- |
|  |  | F | P | F | P | F | P | F | P |
| (Intercept) | *1,192* | 0.00 | 1.000 | 0.00 | 1.000 | 0.00 | 1.000 | 0.00 | 1.000 |
| Particulate components (PC) | *3,24* | 2.06 | 0.132 | 3.82 | **0.023** | 1.08 | 0.377 | 8.41 | **0.001** |
| Earthworms (E) | *1,24* | 0.05 | 0.822 | 0.02 | 0.877 | 10.80 | **0.003** | 0.00 | 0.957 |
| Mesh size (M) | *1,24* | 0.10 | 0.757 | 1.20 | 0.284 | 42.31 | **<0.001** | 52.36 | **<0.001** |
| Time (T) | *4,192* | 133.64 | **<0.001** | 109.38 | **<0.001** | 363.07 | **<0.001** | 588.92 | **<0.001** |
| PC × E | *3,24* | 0.57 | 0.640 | 0.41 | 0.745 | 0.60 | 0.618 | 2.22 | 0.112 |
| PC × M | *3,24* | 0.09 | 0.963 | 1.76 | 0.181 | 1.12 | 0.360 | 4.56 | **0.011** |
| E × M | *1,24* | 0.02 | 0.900 | 2.48 | 0.128 | 1.27 | 0.271 | 0.00 | 0.966 |
| PC × T | *12,192* | 1.70 | 0.069 | 5.16 | **<0.001** | 5.48 | **<0.001** | 8.62 | **<0.001** |
| E × T | *4,192* | 1.79 | 0.133 | 1.19 | 0.318 | 4.64 | **0.001** | 1.16 | 0.329 |
| M × T | *4,192* | 7.75 | **<0.001** | 75.14 | **<0.001** | 16.42 | **<0.001** | 51.48 | **<0.001** |
| PC × E × M | *3,24* | 0.94 | 0.438 | 1.03 | 0.395 | 3.04 | **0.049** | 5.38 | **0.006** |
| PC × E × T | *12,192* | 2.63 | **0.003** | 1.11 | 0.357 | 1.04 | 0.418 | 5.02 | **<0.001** |
| PC × M × T | *12,192* | 2.42 | **0.006** | 1.37 | 0.181 | 3.05 | **0.001** | 9.68 | **<0.001** |
| E × M × T | *4,192* | 1.10 | 0.359 | 0.37 | 0.828 | 0.82 | 0.513 | 1.98 | 0.099 |
| PC × E × M × T | *12,192* | 0.84 | 0.605 | 0.31 | 0.988 | 1.20 | 0.288 | 3.16 | **<0.001** |

**Table S5:**

**F- and P-values of linear mixed-effects models on the effects of particulate components (N, Na, PAHs), earthworms (with, without), mesh size (fine, coarse) and time (70, 140, 210, 280, 365 days) on changes in soil moisture, pH and microbial biomass in the deciduous forest.**

df, numerator and denominator degrees of freedom.

| **Factors** | df | Soil moisture | | Soil pH | | Soil microbial biomass | |
| --- | --- | --- | --- | --- | --- | --- | --- |
|  |  | F | P | F | P | F | P |
| (Intercept) | *1,184* | 28805.0 | **<0.001** | 82940.3 | **<0.001** | 8631.8 | **<0.001** |
| Particulate components (PC) | *3,23* | 0.56 | 0.649 | 11.32 | **<0.001** | 6.47 | **0.002** |
| Earthworms (E) | *1,23* | 1.88 | 0.184 | 1.24 | 0.277 | 6.20 | **0.020** |
| Mesh size (M) | *1,23* | 0.02 | 0.891 | 44.04 | **<0.001** | 185.87 | **<0.001** |
| Time (T) | *4,184* | 8.84 | **<0.001** | 101.45 | **<0.001** | 79.01 | **<0.001** |
| PC × E | *3,23* | 1.10 | 0.370 | 1.80 | 0.175 | 4.96 | **0.008** |
| PC × M | *3,23* | 0.85 | 0.481 | 5.05 | **0.008** | 4.56 | **0.012** |
| E × M | *1,23* | 0.69 | 0.416 | 0.06 | 0.805 | 1.70 | 0.205 |
| PC × T | *12,184* | 0.96 | 0.486 | 9.55 | **<0.001** | 10.48 | **<0.001** |
| E × T | *4,184* | 1.53 | 0.196 | 1.34 | 0.256 | 2.56 | **0.040** |
| M × T | *4,184* | 0.32 | 0.861 | 5.15 | **0.001** | 72.25 | **<0.001** |
| PC × E × M | *3,23* | 1.23 | 0.321 | 0.18 | 0.910 | 0.16 | 0.925 |
| PC × E × T | *12,184* | 1.31 | 0.214 | 1.99 | **0.028** | 2.89 | **0.001** |
| PC × M × T | *12,184* | 0.96 | 0.492 | 4.48 | **<0.001** | 4.21 | **<0.001** |
| E × M × T | *4,184* | 0.93 | 0.447 | 0.79 | 0.534 | 2.57 | **0.039** |
| PC × E × M × T | *12,184* | 1.26 | 0.244 | 1.09 | 0.374 | 4.22 | **<0.001** |

**Table S6:**

**F- and P-values of linear mixed-effects models on the effects of particulate components (N, Na, PAHs), earthworms (with, without), mesh size (fine, coarse) and time (70, 140, 210, 280, 365 days) on changes in soil moisture, pH and microbial biomass in the coniferous forest.**

df, numerator and denominator degrees of freedom.

| **Factors** | df | Soil moisture | | Soil pH | | Soil microbial biomass | |
| --- | --- | --- | --- | --- | --- | --- | --- |
|  |  | F | P | F | P | F | P |
| (Intercept) | *1,192* | 100895.7 | **<0.001** | 229302.9 | **<0.001** | 38275.8 | **<0.001** |
| Particulate components (PC) | *3,24* | 0.30 | 0.822 | 31.11 | **<0.001** | 9.70 | **<0.001** |
| Earthworms (E) | *1,24* | 8.57 | **0.007** | 0.04 | 0.840 | 0.22 | 0.640 |
| Mesh size (M) | *1,24* | 2.10 | 0.160 | 3.22 | 0.085 | 17.37 | **<0.001** |
| Time (T) | *4,192* | 7.75 | **<0.001** | 29.89 | **<0.001** | 368.21 | **<0.001** |
| PC × E | *3,24* | 0.07 | 0.975 | 0.42 | 0.742 | 1.44 | 0.257 |
| PC × M | *3,24* | 0.31 | 0.815 | 6.19 | **0.003** | 2.59 | 0.077 |
| E × M | *1,24* | 0.47 | 0.499 | 0.84 | 0.368 | 2.51 | 0.126 |
| PC × T | *12,192* | 1.37 | 0.181 | 13.07 | **<0.001** | 3.92 | **<0.001** |
| E × T | *4,192* | 6.99 | **<0.001** | 3.78 | **0.006** | 0.68 | 0.606 |
| M × T | *4,192* | 0.41 | 0.803 | 2.62 | **0.036** | 14.08 | **<0.001** |
| PC × E × M | *3,24* | 1.26 | 0.309 | 1.59 | 0.217 | 0.65 | 0.588 |
| PC × E × T | *12,192* | 1.75 | 0.059 | 0.98 | 0.466 | 1.46 | 0.143 |
| PC × M × T | *12,192* | 0.76 | 0.687 | 2.30 | **0.009** | 1.57 | 0.105 |
| E × M × T | *4,192* | 0.21 | 0.933 | 1.57 | 0.183 | 1.66 | 0.162 |
| PC × E × M × T | *12,192* | 1.71 | 0.068 | 1.34 | 0.197 | 1.50 | 0.127 |

Figures

**Figure S1**


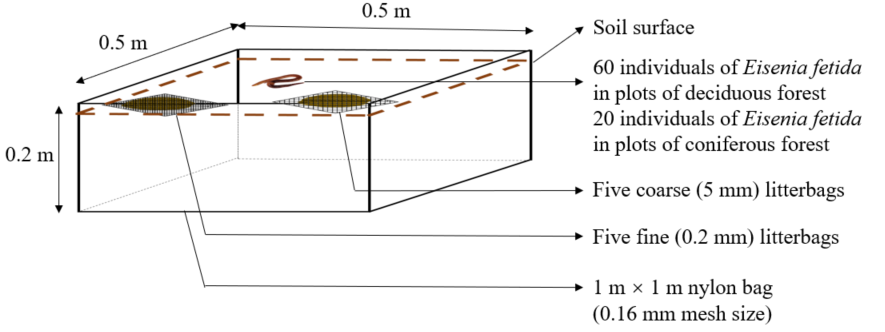


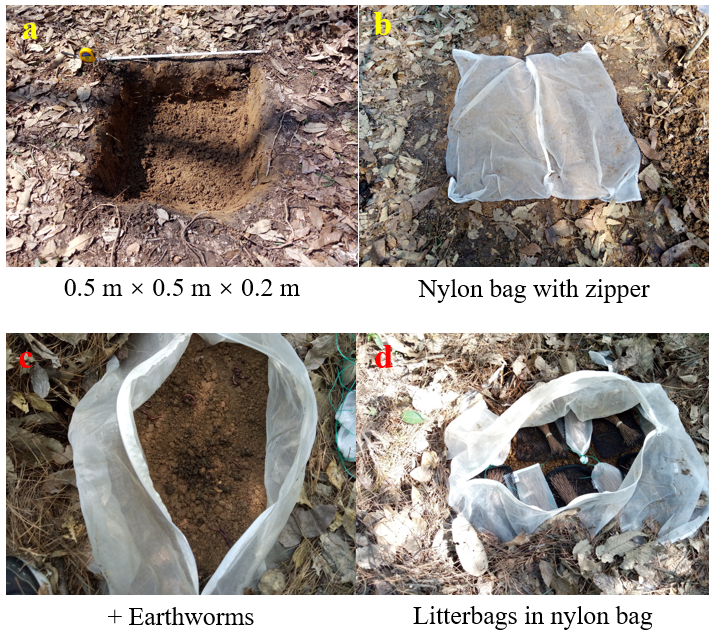


**Figure** **S1:**

**Experimental design (top scheme) and set-up of the mesocosms.**

(a) excavation of the soil pit of 0.5 m × 0.5 m × 0.2 m; (b) refilled pits lined and closed with zip log mesh (0.16 mm) bags (mesocosms); (c) addition of *Eisenia fetida* individuals to the mesocosms; (d) placement of litterbags into the mesocosms.

**Figure S2**


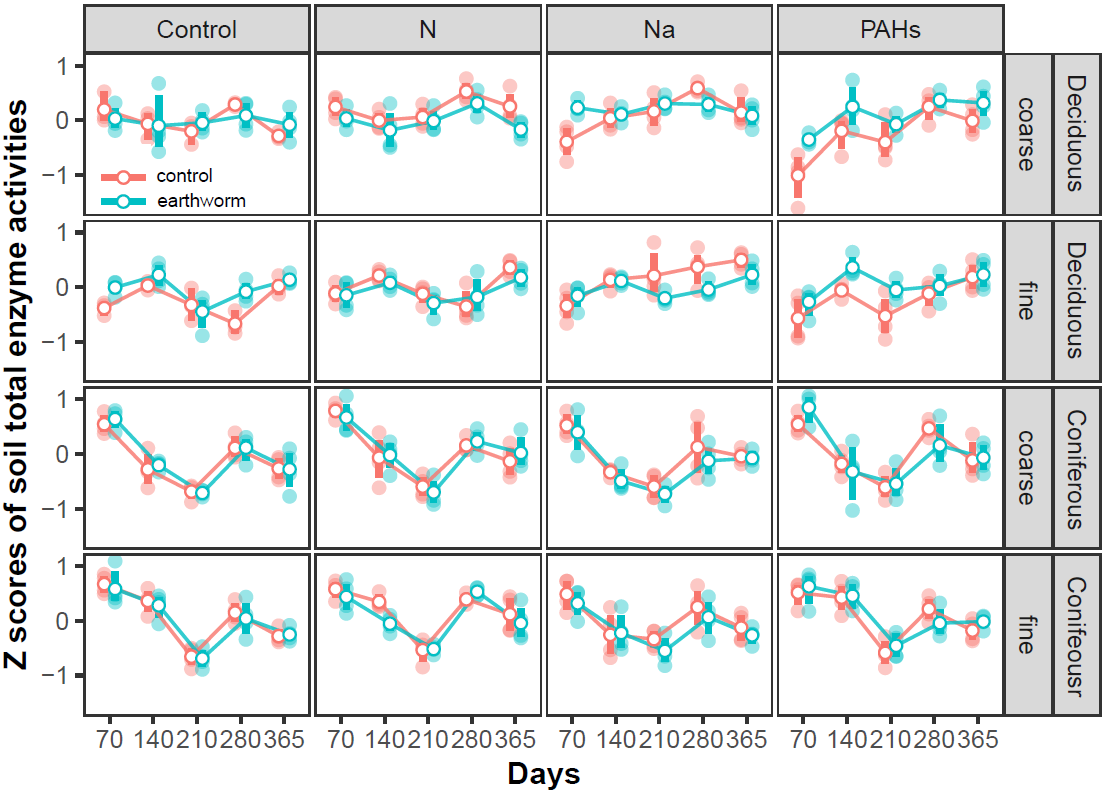


**Figure S2:**

**Changes in Z scores of soil total enzyme activities with time (70, 140, 210, 280, 365 days) as affected by different types of particulate components (Control, N, Na, PAHs) and earthworms (with, without) in the soil underneath the coarse and fine mesh size litterbags in deciduous and coniferous forests.**

Means ± SE, n = 4.

**Figure S3**


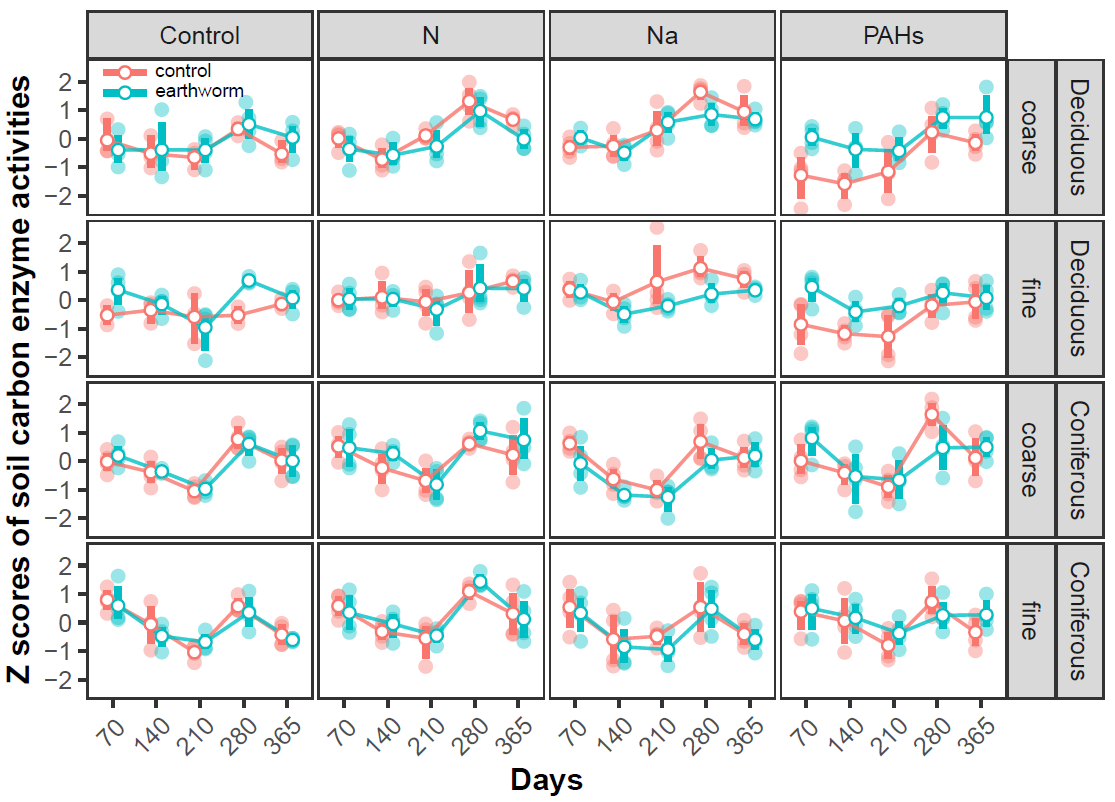


**Figure S3:**

**Changes in Z scores of soil carbon enzyme activities (β-1,4-glucosidase, β-1,4-xylosidase and cellobiohydrolase) with time (70, 140, 210, 280, 365 days) as affected by different types of particulate components (Control, N, Na, PAHs) and earthworms (with, without) in the soil underneath the coarse and fine mesh size litterbags in deciduous and coniferous forests.**

Means ± SE, n = 4.

**Figure S4**


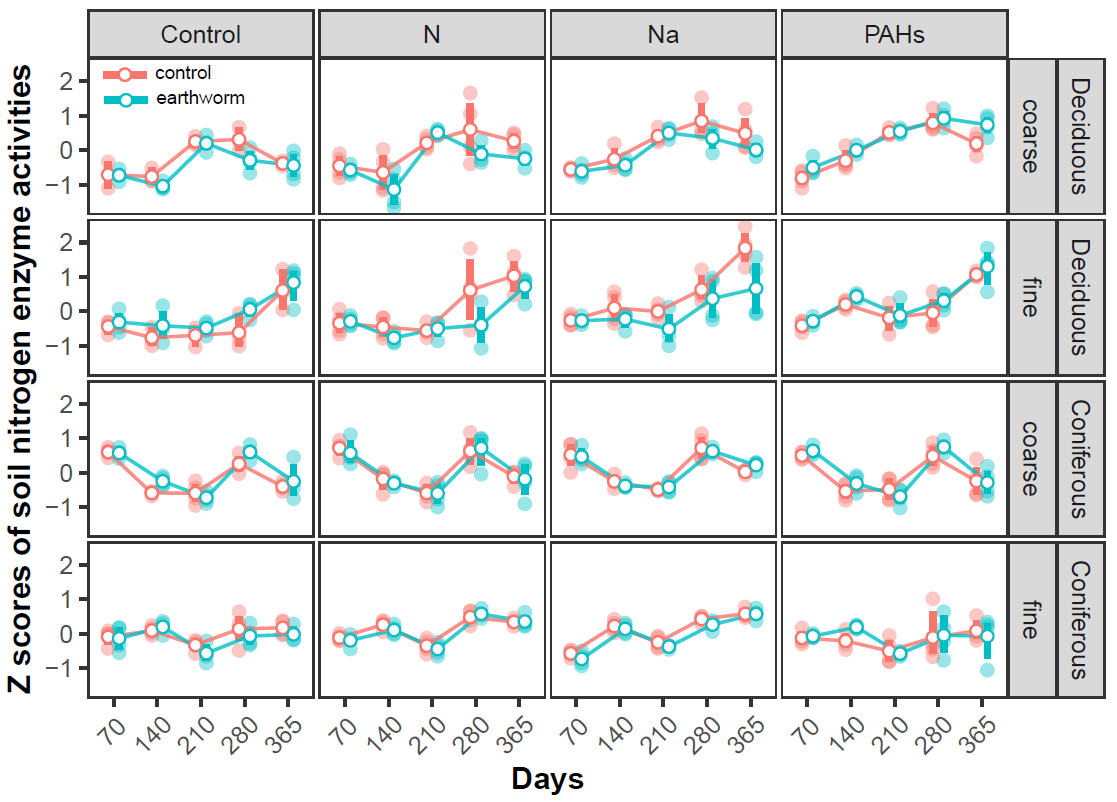


**Figure S4:**

**Changes in Z scores of soil nitrogen enzyme activities (nitrate reductase and urease) with time (70, 140, 210, 280, 365 days) as affected by different types of particulate components (Control, N, Na, PAHs) and earthworms (with, without) in the soil underneath the coarse and fine mesh size litterbags in deciduous and coniferous forests.**

Means ± SE, n = 4.

**Figure S5**


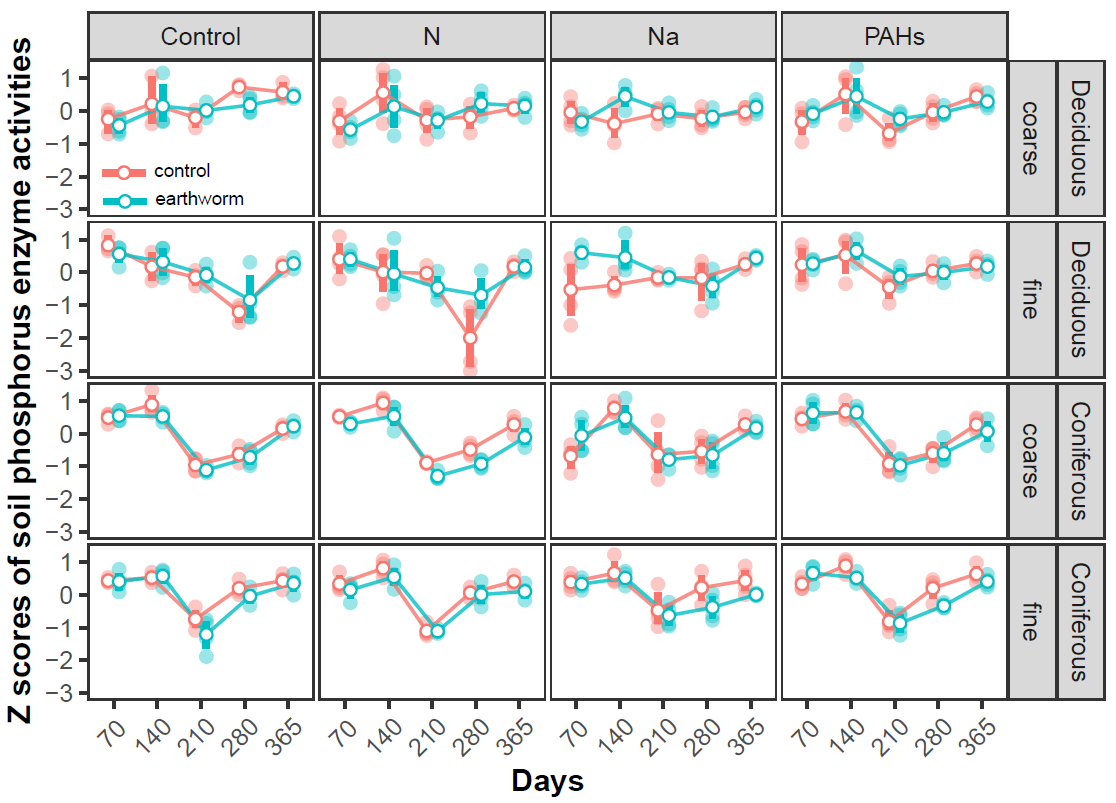


**Figure S5:**

**Changes in Z scores of soil phosphorus enzyme activities (acid and alkaline phosphatases) with time (70, 140, 210, 280, 365 days) as affected by different types of particulate components (Control, N, Na, PAHs) and earthworms (with, without) in the soil underneath the coarse and fine mesh size litterbags in deciduous and coniferous forests.**

Means ± SE, n = 4.

**Figure S6**


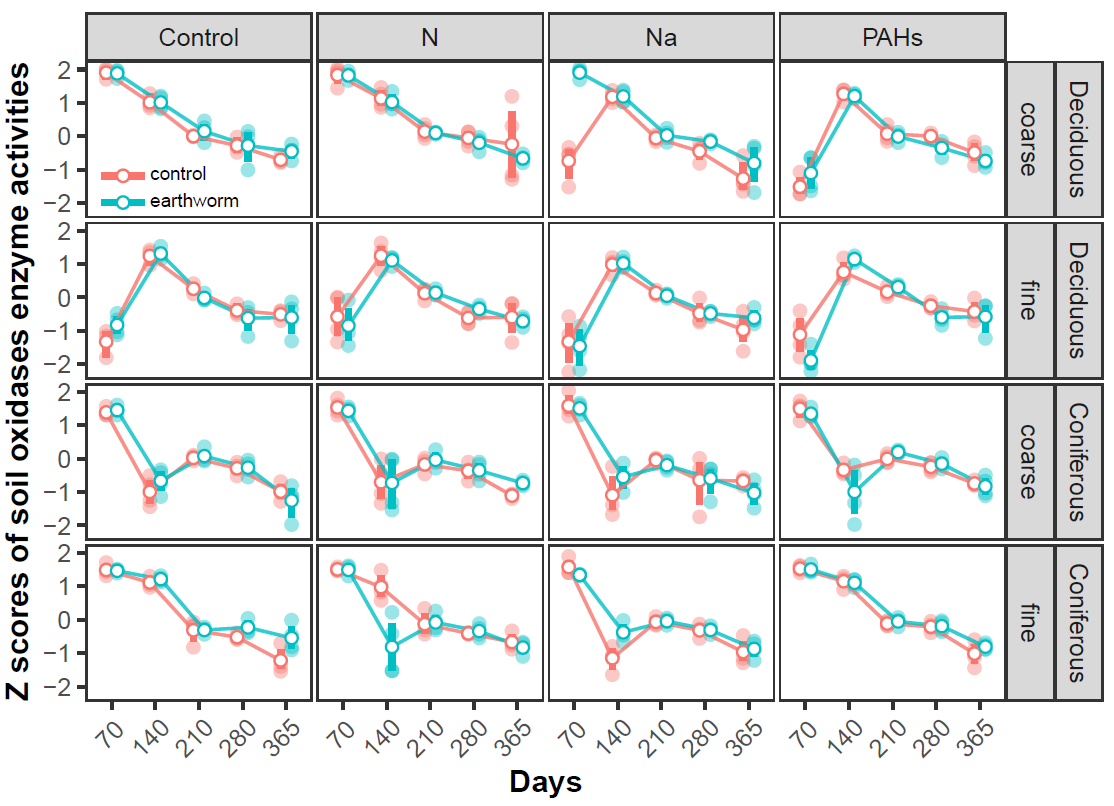


**Figure S6:**

**Changes in Z scores of soil oxidase activities (peroxidase and polyphenol oxidase) with time (70, 140, 210, 280, 365 days) as affected by different types of particulate components (Control, N, Na, PAHs) and earthworms (with, without) in the soil underneath the coarse and fine mesh size litterbags in deciduous and coniferous forests.**

Means ± SE, n = 4.

**Figure S7**


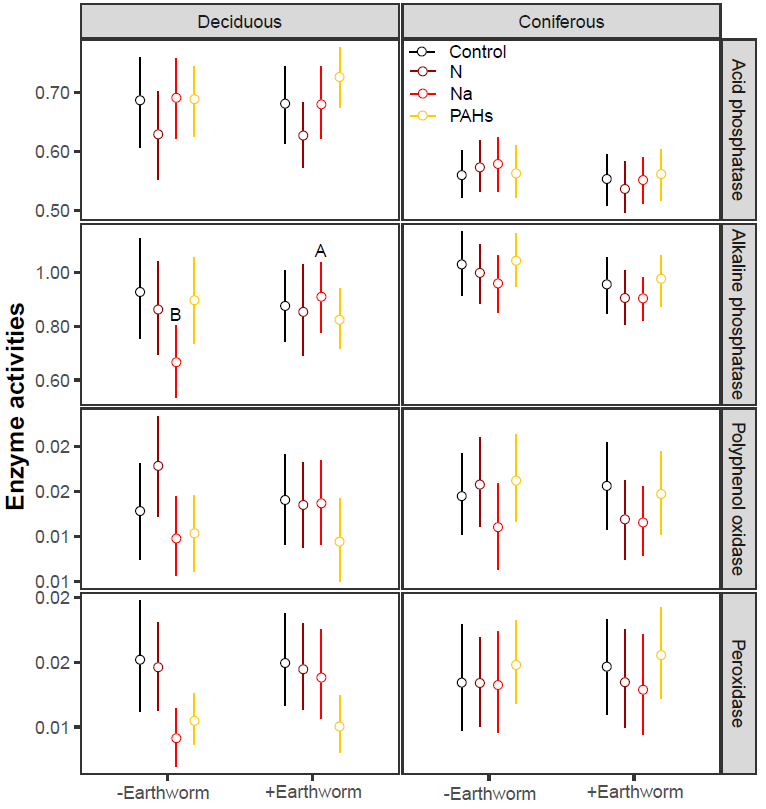


**Figure S7:**

**Changes in extracellular phosphorus enzyme and oxidase activities (acid phosphatase, alkaline phosphatase, polyphenol oxidase, peroxidase) as affected by different types of particulate components (Control, N, Na, PAHs) and earthworms (without, with) in the soil underneath the litterbags in deciduous and coniferous forests.**

Means ± SE, values were averaged across sampling dates (70, 140, 210, 280, 365 day) and mesh size (coarse and fine), n = 40; capital letters indicated the difference in enzyme activities in earthworm treatments (without, with).

**Figure S8**


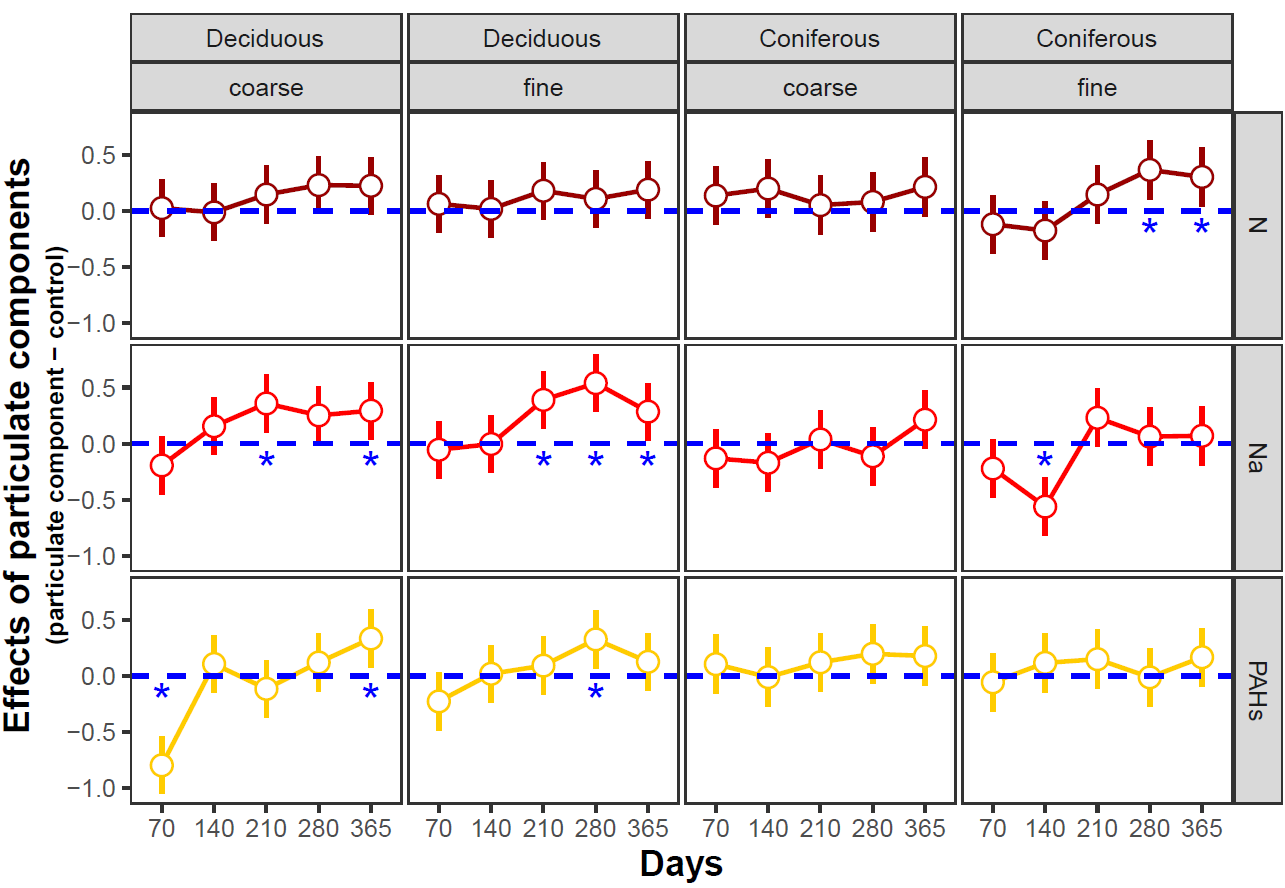


**Figure S8:**

**Changes in estimates of Z scores of soil total enzyme activities with time (70, 140, 210, 280, 365 days) as affected by different types of particulate components (N, Na, PAHs) and mesh sizes of litterbags (coarse and fine) in deciduous and coniferous forests.**

Means with 95% confidence intervals; effect sizes were averaged across earthworm treatments (with and without), n = 8. Asterisks indicate significant differences to the control (P < 0.05).

**Figure S9**


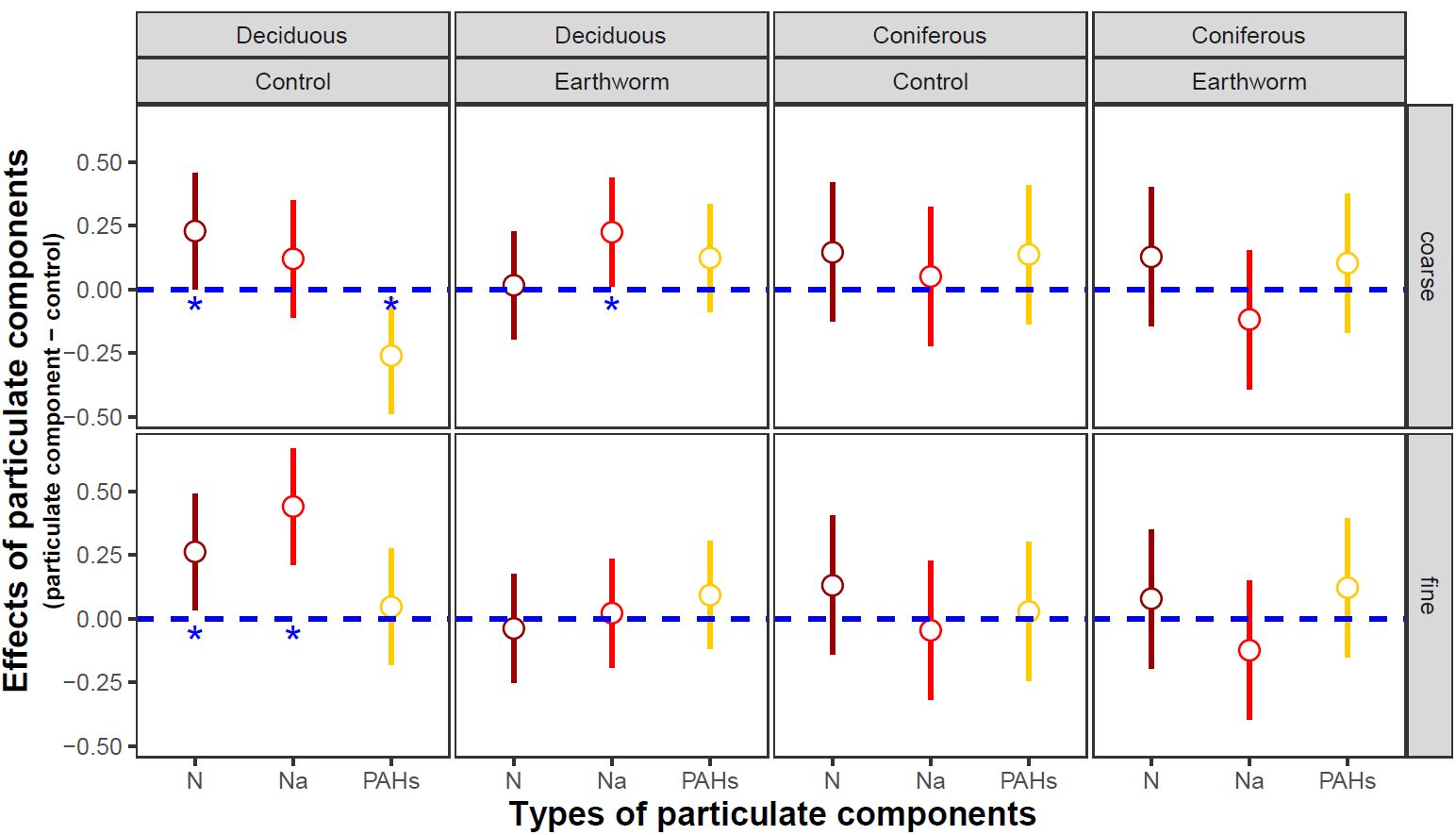


**Figure S9:**

**Changes in estimates of Z scores of soil total enzyme activities as affected by different types of particulate components (N, Na, PAHs), earthworms (with and without) and mesh sizes of litterbags (coarse and fine) in deciduous and coniferous forests.**

Means with 95% confidence intervals; effect sizes were averaged across five times (70, 140, 210, 280, 365 days), n = 20. Asterisks indicate significant differences to the control (P < 0.05).

**Figure S10**


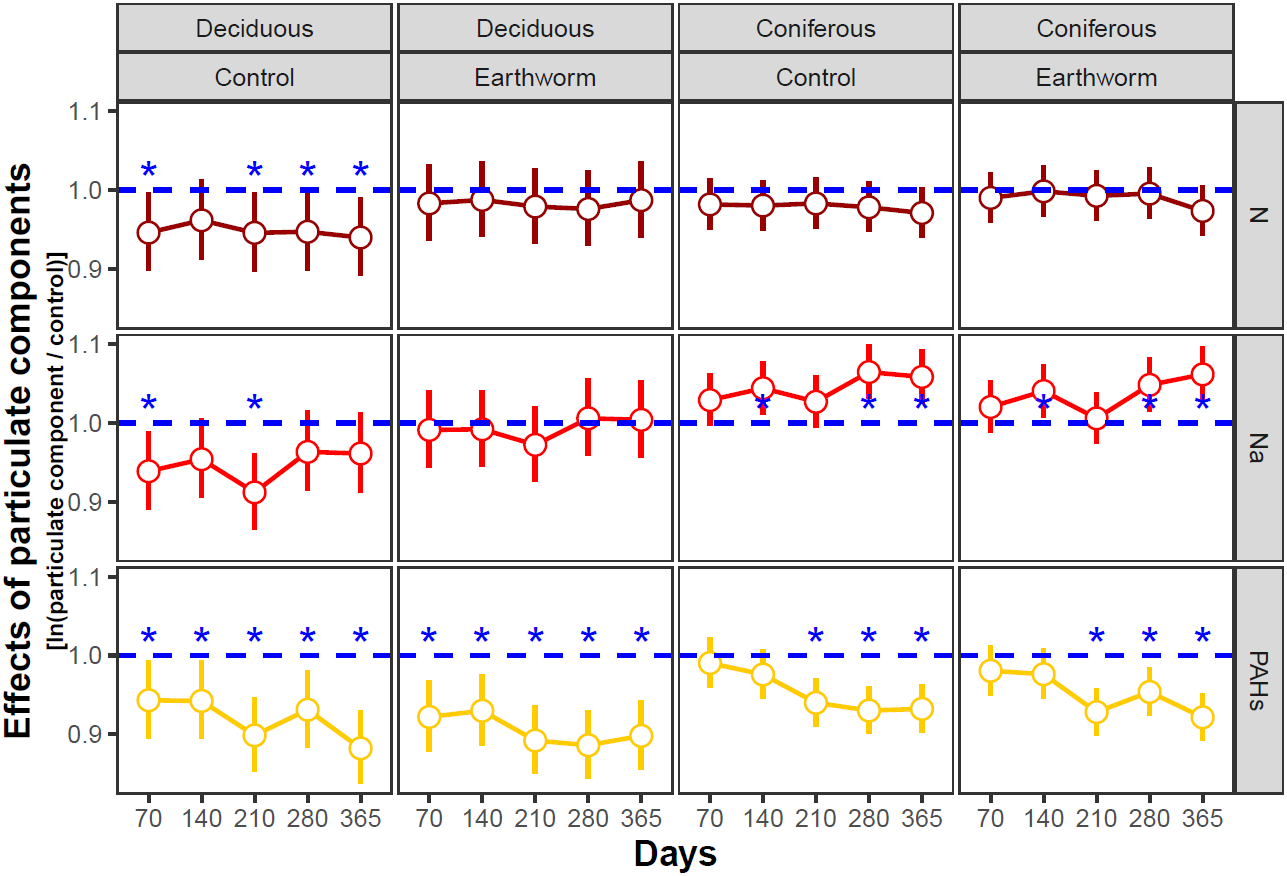


**Figure S10:**

**Changes in estimates of soil pH with time (70, 140, 210, 280, 365 days) as affected by different types of particulate components (N, Na, PAHs) and earthworms (without and with) in deciduous and coniferous forests.**

Means with 95% confidence intervals; effect sizes were averaged across mesh size (coarse and fine), n = 8. Asterisks indicate significant differences to the control (P < 0.05).

**Figure S11**


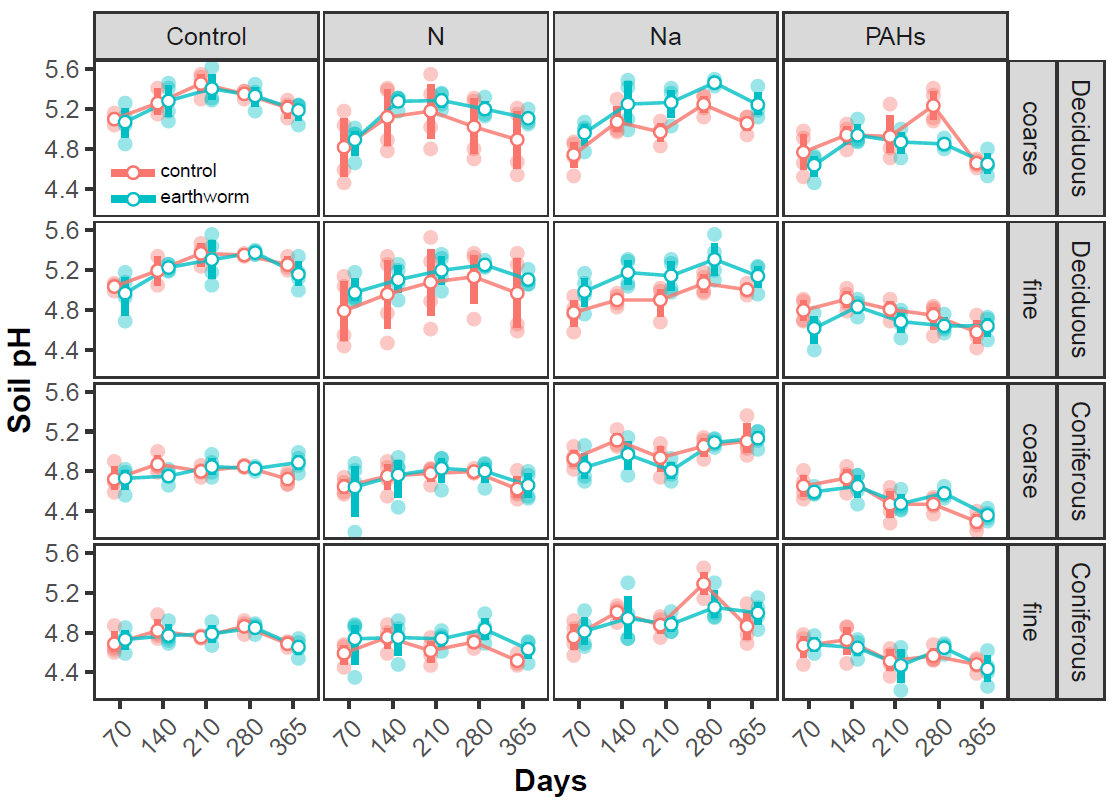


**Figure S11:**

**Changes in soil pH with time (70, 140, 210, 280, 365 days) as affected by different types of particulate components (Control, N, Na, PAHs) and earthworms (with, without) in the soil underneath the coarse and fine mesh size litterbags in deciduous and coniferous forests.**

Means ± SE, n = 4.

**Figure S12**


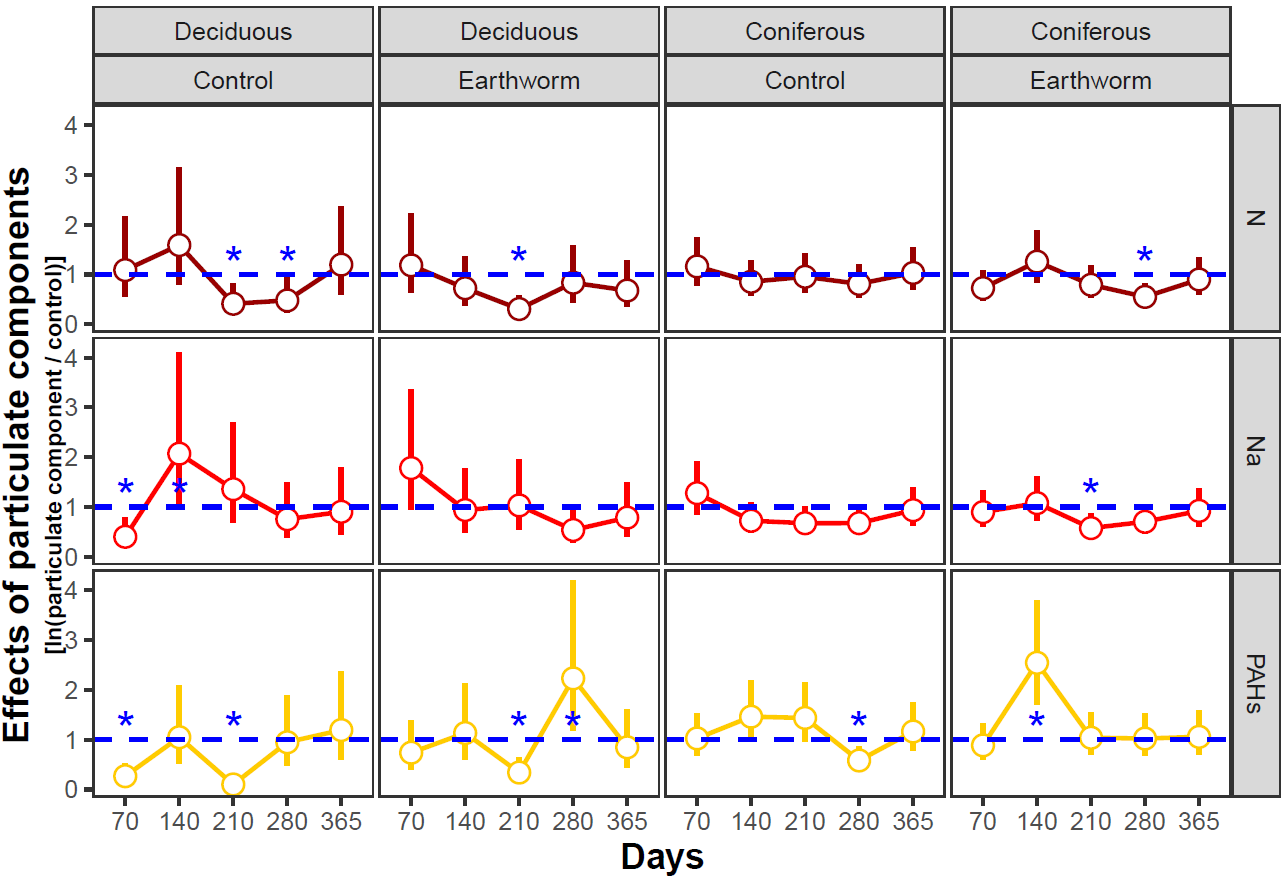


**Figure S12:**

**Changes in estimates of soil microbial biomass with time (70, 140, 210, 280, 365 days) as affected by different types of particulate components (N, Na, PAHs) and earthworms (without and with) in deciduous and coniferous forests.**

Means with 95% confidence intervals; effect sizes were averaged across mesh size (coarse and fine), n = 8. Asterisks indicate significant differences to the control (P < 0.05).

**Figure S13**


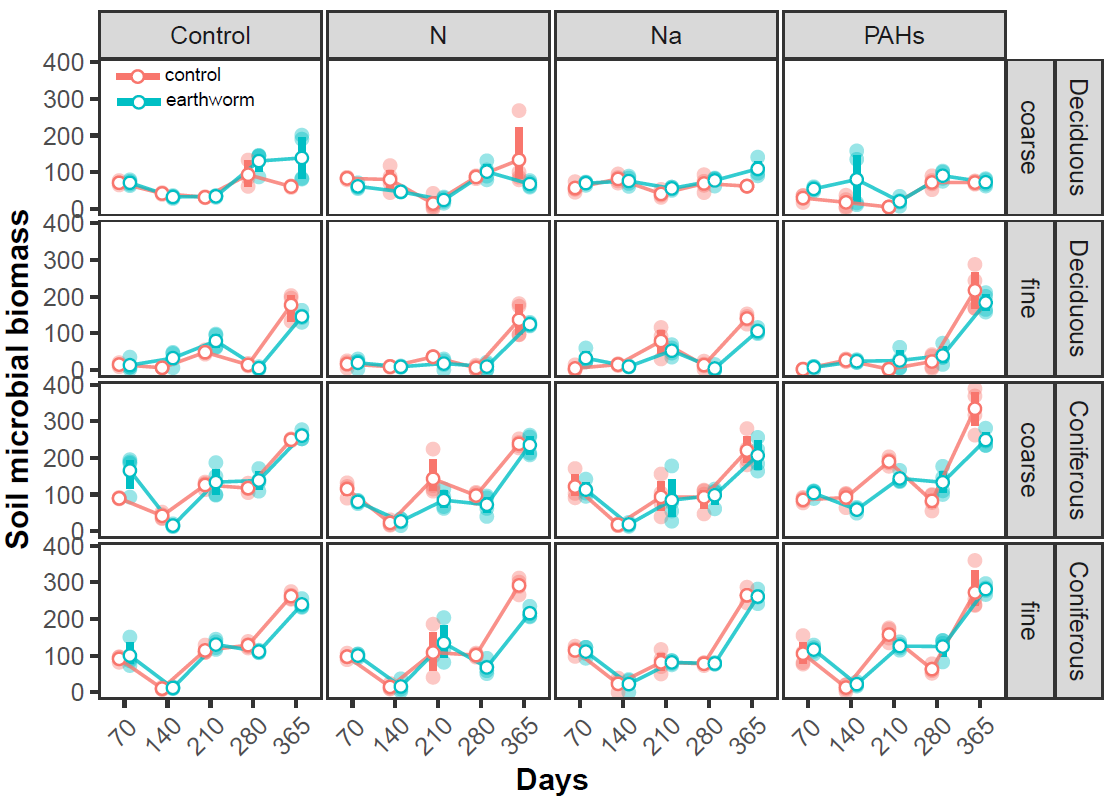


**Figure S13:**

**Changes in soil microbial biomass (SIR) with time (70, 140, 210, 280, 365 days) as affected by different types of particulate components (Control, N, Na, PAHs) and earthworms (with, without) in the soil underneath the coarse and fine mesh size litterbags in deciduous and coniferous forests.**

Means ± SE, n = 4. The units of soil microbial biomass were expressed as the amount of CO_2_ produced by 1 g fresh soil (60% water holding capacity) with 1 mL aqueous glucose solution (10 mg glucose g^-1^ soil) in 60 min, i.e. ppm g^-1^ soil h^-1^.

**Figure S14**


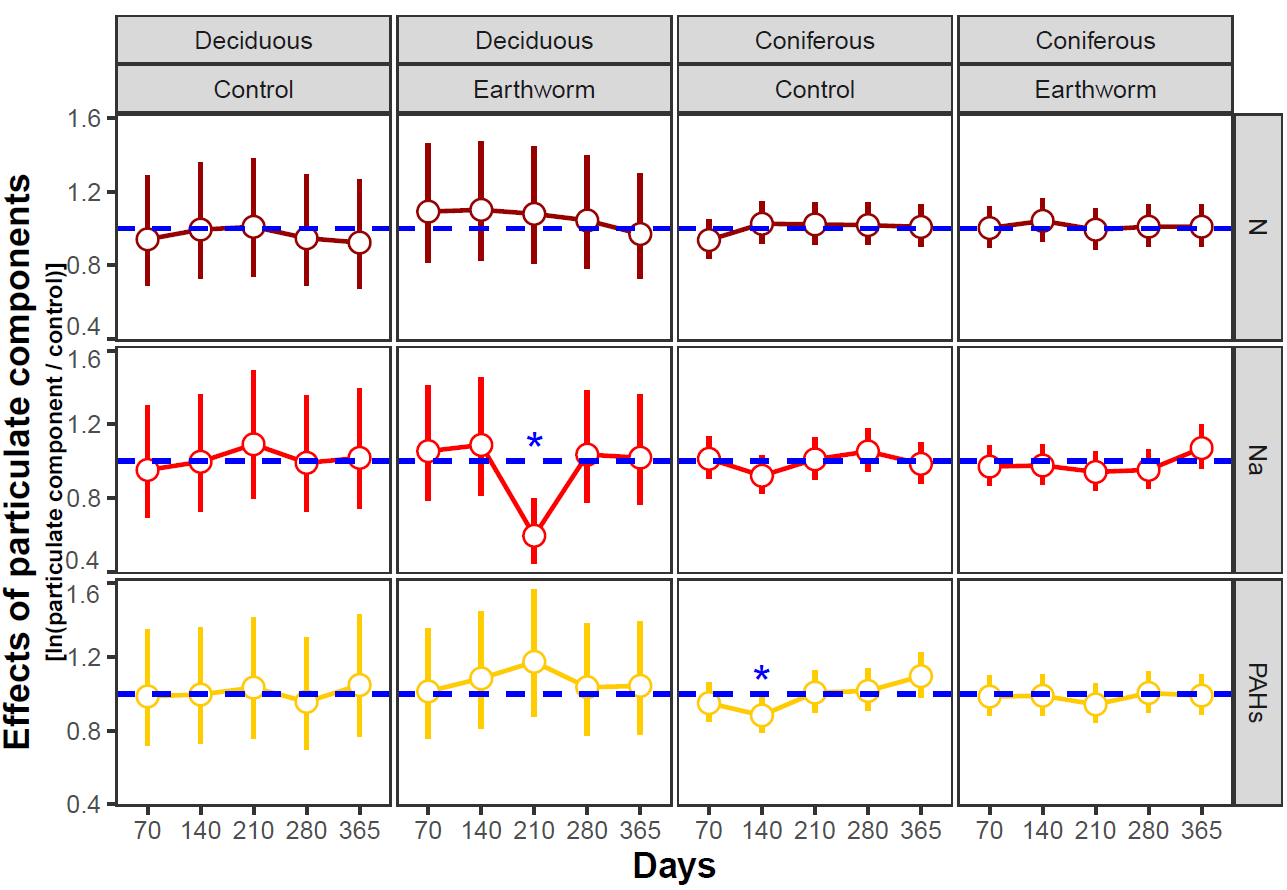


**Figure 14:**

**Changes in estimates of soil moisture with time (70, 140, 210, 280, 365 days) as affected by different types of particulate components (N, Na, PAHs) and earthworms (with and without) in deciduous and coniferous forests.**

Means with 95% confidence intervals; effect sizes were averaged across mesh size (coarse and fine), n = 8; asterisks indicate significant differences to the control (P < 0.05).

**Figure S15**


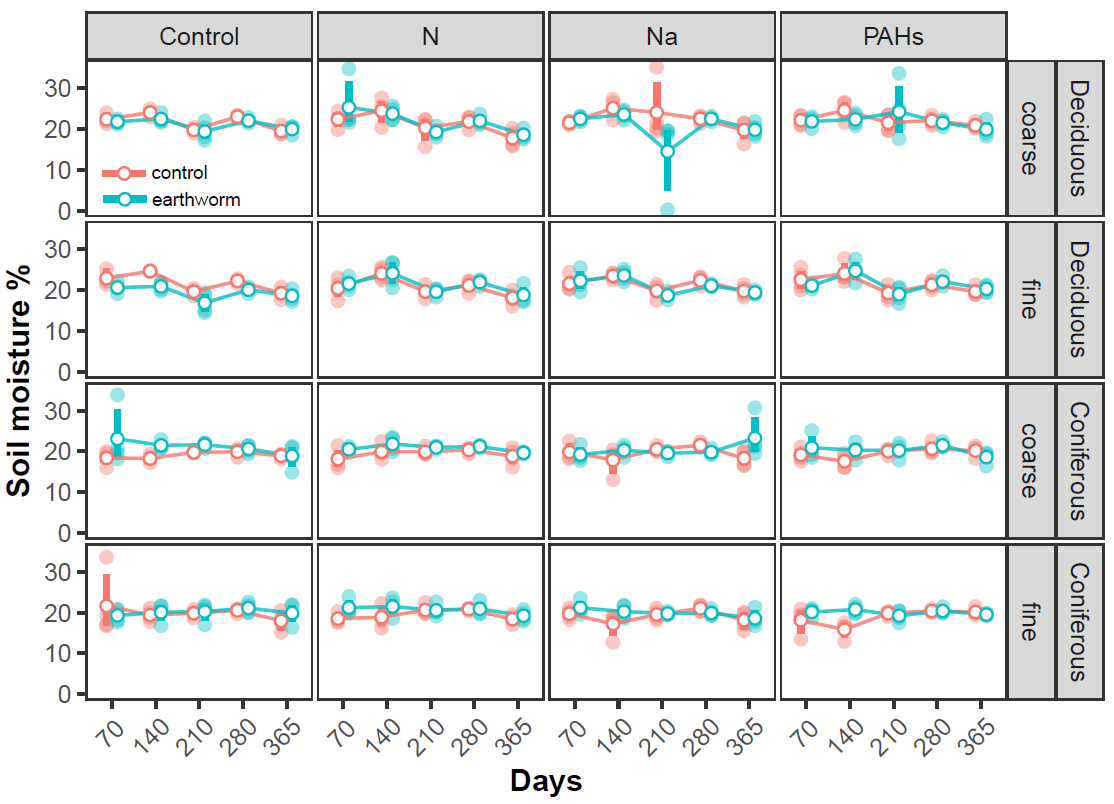


**Figure S15:**

**Changes in soil moisture with time (70, 140, 210, 280, 365 days) as affected by different types of particulate components (Control, N, Na, PAHs) and earthworms (with, without) in the soil underneath the coarse and fine mesh size litterbags in deciduous and coniferous forests.**

Means ± SE, n = 4.

**Figure S16**


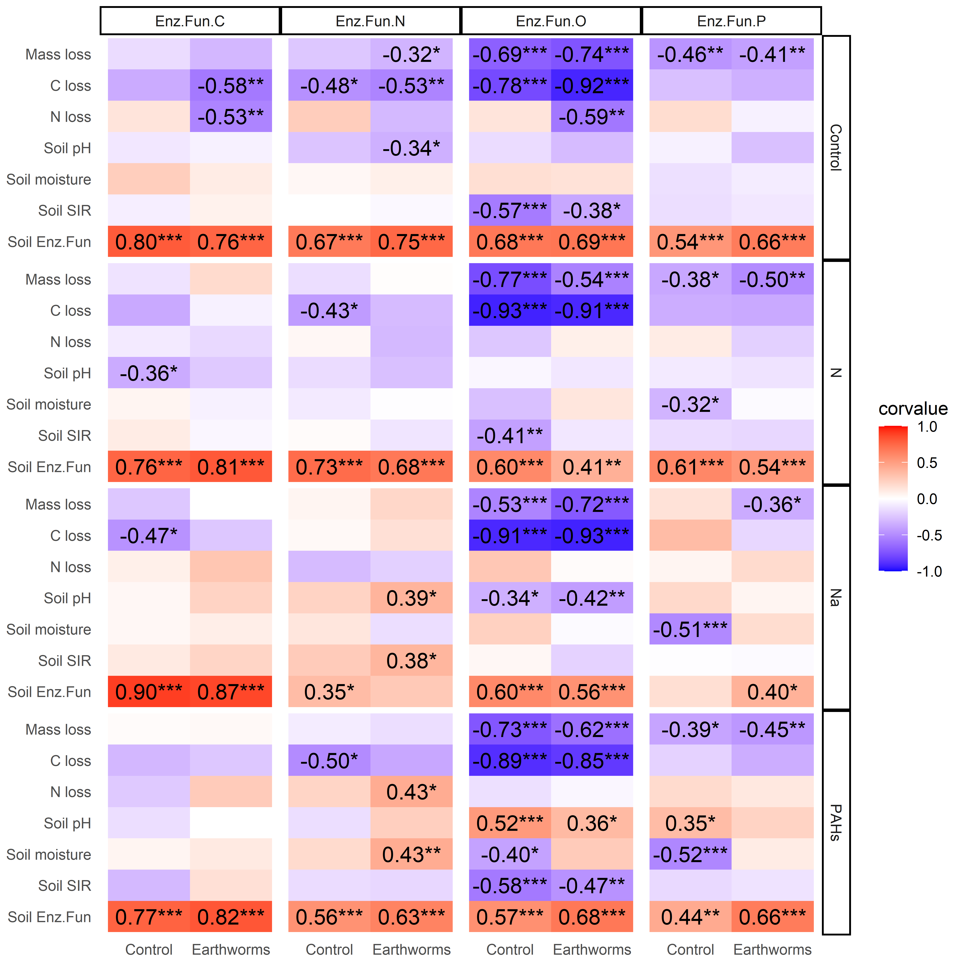


**Figure S16:**

**Correlation matrix of different types of soil enzyme functions (C, N, O, P) with litter decomposition (mass loss, C loss, N loss) and soil properties (pH, moisture, SIR, total enzyme function) in the different particulate components (control, N, Na, PAHs) and earthworm treatments (with and without) in the coniferous forest.**

Enz.Fun.C, Enz.Fun.N, Enz.Fun.O, Enz.Fun.P, Soil Enz.Fun refer to enzyme functions of carbon, nitrogen, oxidase, phosphorus and total soil enzymatic function, respectively. Mass loss, C loss, N loss refer to percentage changes during decomposition of *Pinus massoniana* litter. Corvalue refers to the Pearson correlation coefficient and is given if the p-value was < 0.05; *, P ≤ 0.05; **, P ≤ 0.01; ***, P ≤ 0.001.
